# Computational Method for Classification of Avian Influenza A Virus Using DNA Sequence Information and Physicochemical Properties

**DOI:** 10.3389/fgene.2021.599321

**Published:** 2021-01-28

**Authors:** Fahad Humayun, Fatima Khan, Nasim Fawad, Shazia Shamas, Sahar Fazal, Abbas Khan, Arif Ali, Ali Farhan, Dong-Qing Wei

**Affiliations:** ^1^State Key Laboratory of Microbial Metabolism, Department of Bioinformatics and Biological Statistics, School of Life Sciences and Biotechnology, Shanghai Jiao Tong University, Shanghai, China; ^2^Department of Bioinformatics and Biosciences, Capital University of Science and Technology, Islamabad, Pakistan; ^3^Poultry Research Institute, Rawalpindi, Pakistan; ^4^Department of Zoology, University of Gujrat, Gujrat, Pakistan; ^5^Department of Bioinformatics and Biotechnology, Government College University Faisalabad, Faisalabad, Pakistan

**Keywords:** Avian influenza A Virus, k-gram, discrete wavelet transform, multivariate mutual information, decision tree, K-nearest neighbor, Naïve Bayes, support vector machine

## Abstract

Accurate and fast characterization of the subtype sequences of Avian influenza A virus (AIAV) hemagglutinin (HA) and neuraminidase (NA) depends on expanding diagnostic services and is embedded in molecular epidemiological studies. A new approach for classifying the AIAV sequences of the HA and NA genes into subtypes using DNA sequence data and physicochemical properties is proposed. This method simply requires unaligned, full-length, or partial sequences of HA or NA DNA as input. It allows for quick and highly accurate assignments of HA sequences to subtypes H1–H16 and NA sequences to subtypes N1–N9. For feature extraction, k-gram, discrete wavelet transformation, and multivariate mutual information were used, and different classifiers were trained for prediction. Four different classifiers, Naïve Bayes, Support Vector Machine (SVM), K nearest neighbor (KNN), and Decision Tree, were compared using our feature selection method. This comparison is based on the 30% dataset separated from the original dataset for testing purposes. Among the four classifiers, Decision Tree was the best, and Precision, Recall, F1 score, and Accuracy were 0.9514, 0.9535, 0.9524, and 0.9571, respectively. Decision Tree had considerable improvements over the other three classifiers using our method. Results show that the proposed feature selection method, when trained with a Decision Tree classifier, gives the best results for accurate prediction of the AIAV subtype.

## Introduction

The influenza virus belongs to the family of Orthomyxoviridae, comprising a viral envelope containing glycol protein forms with a central nucleus. There is a viral genome and other proteins in the main nucleus. Almost eleven proteins are encoded in the viral genome by eight pieces of single-stranded negative-sense RNA. There are three influenza virus forms: A, B, and C (Kawaoka, 2006). Avian influenza A Virus (AIAV) remains a significant threat to human and animal health (Guan et al., 2010; Suarez, 2010). To track the distribution and examine developmental patterns of AIAVs, expensive testing systems and detailed molecular epidemiological studies are needed. The better phylogenetic clustering and subtyping of AIAVs is focused on the genetic and antigenic properties of the proteins and genomes of the immune-dominant viral hemagglutinin (HA) and neuraminidase (NA) in at least 16 HA and 9 NA subtypes at present (Suarez, 2010). Events of re-assortment and rapid HA gene mutation driven by immune-selective pressure cause antigenic drift and antigenic shift of the affected viruses, respectively (World Health Organization, 2011; Mak et al., 2012). Furthermore, these processes result in a rapid progression of phylogenetic diversification that questions the classification of subtypes and clusters needed for molecular epidemiological analysis (Altschul et al., 1990).

Hemagglutination-inhibition (HI) assay and neuraminidase-inhibition (NI) assay are the standard ways of determining the Avian Influenza virus subtype of HA and NA segments capable of distinguishing antigenic variations even from the same subtype. Nevertheless, as described in Pederson (2008), the inventory of reference reagents must identify antigenically distinct influenza viruses and antibody specificities from multiple lines of a single hemagglutinin subtype. It requires extensive laboratory assistance to produce and refine reagents while dealing with uncharacterized viruses or antibody subtypes. It is much cheaper and faster to use machine learning approaches to forecast virus history, but it can usually still deliver high levels of accuracy (Pederson, 2008).

A variety of machine learning methods classify biological information relationships or connections, band similar genetic components, and interpret and forecast diseases (Salzberg et al., 1998; Yuan et al., 2003; Sami and Takahashi, 2005). Machine learning includes the automated development of data models and the use of these systems for automatic inference and prediction (Langley, 1996). Since it is about classifying viral strains, recognizing the role of specific positions, and modeling them for future prediction, which addresses viral influenza analysis, machine learning techniques have a lot to offer. Biological evidence has many characteristics, complicated relationships, and often lacks a clear explanation behind it. Machine learning techniques work well with this information due to their ability to tackle randomness, software noise volatility, and generalizations.

To follow evolutionary patterns and spreading paths, methods for accurate identification of the individual clusters are essential. The WHO H5N1 Evolution Working Group developed a standardized nomenclature scheme based on the genetic resemblance between the entire usable HA sequences. It was the primary step toward the classification of highly pathogenic avian influenza virus (HPAIV). This annotation offers the possibility of separating HPAIV into different classes and lines. The group recently defined 32 clades as a hierarchical tree based on genetic resemblance (Pleschka, 2012). Sadly, this process does not have a rapid generic procedure for assigning freshly formed and uncategorized HA sequences. Common approaches to the assignment of new sequences while computing lengthy phylogenetic methods consist of a simple search for BLAST (Edgar, 2004) against a collection of categorized HA sequences or the development of a smaller phylogenetic tree of assigned and unclassified sequences.

Nonetheless, such approaches are inhomogeneous and cumbersome. There is no guideline for selecting the BLAST reference list or the phylogenetic tree, nor a clear standard for assigning sequences to a particular subtype or clade. The “Highly Pathogenic H5N1 Clade Classification Tool” (CT) has recently been introduced as a free web tool by the Influenza Research Database (IRD^1^). The IRD web tool is based on phylogeny but maintains the tree of sequences already classified (Squires et al., 2012). Outside of the creation of the IRD, to follow a different strategy, ClassyFlu was developed to focus on Hidden Markov models with a discriminatively trained profile. ClassyFlu’s assignment capacity is tested and contrasted to IRD-CT and other methods of classification. ClassyFlu quickly assigns HA and NA sequences to the related subtypes and also correctly integrates H5 sequences, identical to IRD-CT, into the H5N1 clade scheme (Van der Auwera et al., 2014). These approaches were used to predict the Influenza A virus affecting humans and have some limitations. Previously, surveillance tools for predicting the spread of avian influenza virus was developed (Yousefinaghani et al., 2020). Furthermore, a machine learning method was also devised to predict global reservoirs for low pathogenic avian influenza virus using big data (Gulyaeva et al., 2020) but there was no specific strategy to classify only Avian Influenza A virus subtypes, so there was a need to develop a method.

To suggest an efficient statistical approach for the prediction of the Avian Influenza A virus subtype, we merged three methods of extraction of features: k-gram, discrete wavelet transformation (DWT), and multivariate mutual information (MMI). The k-gram can get the frequency characteristics of an item in a sequence and has been used related to computational biology (Wong et al., 2013). The association details between two nucleotides can be examined by MMI (Ding et al., 2016). The frequency and location information can be captured by DWT (Shen et al., 2017). We can train a classifier to achieve the Avian Influenza Virus subtype’s best performance and accurate prediction with these features. We also analyzed the significance of each of these methods. The results of four different classifiers, Decision Tree, Support Vector Machine (SVM), Naïve Bayes, and K nearest neighbor (KNN), were compared using our feature selection method. Decision Tree had significant improvements as compared to the other three classifiers.

## Materials and Methods

### Hemagglutinin (HA) Subtypes

Our classification method’s parameter training requires the class definition to which the later input sequences are assigned. Sequences and their predefined identification from the NCBI Influenza Virus Tool http:/www.ncbi.nlm.nih.gov/genomes/FLU were used, although this could be achieved *de novo* in theory by sequence clustering or using phylogenetic analysis. A maximum of 16 categories was used, including H1–H16 subtypes of avian influenza virus. The training data contains all HA gene sequences that were at least 1,600 bp long and published through December 2019. In particular, 26,586 sequences were obtained for the classifier. Data was distributed as 70% for training and 30% for testing for the classifier (Supplementary Table 1).

### Neuraminidase (NA) Subtypes

Based on all usable non-redundant NA sequences reported in the NCBI Influenza Virus Tool http:/www.ncbi.nlm.nih.gov/genomes/FLU by September 2019, we picked for training collection. Short sequences and sequences comprising unclear nucleotides were excluded. Based on sequence similarities, a representative subset was chosen for each NA subtype N1–N9 of avian influenza virus. In total, 20,690 sequences were selected for the classifier, and the same criteria of distribution of 70% data for training and 30% for testing were adopted. The training data contains all NA gene sequences that were collected and were at least l 1,300 bp long (Supplementary Table 2).

### Physicochemical Properties

Nucleotides have 16 2-permutations, that is, AA, AT,…, AC. According to an earlier research study, each permutation has six physicochemical properties (Twist, Tilt, Turn, Move, Slip, and Rise) linked to its physical structure. The unique values for the six new structural physical properties revised by Goni et al. are summarized in Table 1 (Goñi et al., 2007).

**TABLE 1:** Six physical structural properties and its values.

2-Nucleotide	Twist	Tilt	Roll	Shift	Slide	Rise
***AA***	0.026	0.038	0.020	1.69	2.26	7.65
***AC***	0.036	0.038	0.023	1.32	3.03	8.93
***AG***	0.031	0.037	0.019	1.46	2.03	7.08
***AT***	0.033	0.036	0.022	1.03	3.83	9.07
***CA***	0.016	0.025	0.017	1.07	1.78	6.38
***CC***	0.026	0.042	0.019	1.43	1.65	8.04
***CG***	0.014	0.026	0.016	1.08	2.00	6.23
***CT***	0.031	0.037	0.019	1.46	2.03	7.08
***GA***	0.025	0.038	0.020	1.32	1.93	8.56
***GC***	0.025	0.036	0.026	1.20	2.61	9.53
***GG***	0.026	0.042	0.019	1.43	1.65	8.04
***GT***	0.036	0.038	0.023	1.32	3.03	8.93
***TA***	0.017	0.018	0.016	0.72	1.20	6.23
***TC***	0.025	0.038	0.020	1.32	1.93	8.56
***TG***	0.016	0.025	0.017	1.07	1.78	6.38
***TT***	0.026	0.038	0.020	1.69	2.26	7.65

One length (*l* + 1) nucleotide sequence *L* = *N*_1_*N*_2_….*N*_*l*_*N*_*l* + 1_ that are based on physical structural properties can be transformed into one *l*×6matrix PC as in Equation (1).

(1) $$P\,C=\left\{\begin{array}{cccc} P\,M\,\left[N_{1}\,N_{2},P_{1}\right] & P\,M\,\left[N_{1}\,N_{2},P_{2}\right] & \ldots & P\,M\,\left[N_{1}\,N_{2},P_{6}\right]\\ P\,M\,\left[N_{2}\,N_{3},P_{1}\right] & P\,M\,\left[N_{2}\,N_{3},P_{2}\right] & \ldots & P\,M\,\left[N_{2}\,N_{3},P_{6}\right]\\ . & . & .\\ . & . & .\\ . & . & .\\ P\,M\,\left[N_{l}\,N_{l+1},P_{1}\right] & P\,M\,\left[N_{l}\,N_{l+1},P_{2}\right] & \ldots & P\,M\,\left[N_{l}\,N_{l+1},P_{6}\right] \end{array}\right\}$$

Where *PM*[*N*_*i*_*N*_*i* + 1_,*P*_*j*_] is the structural physical properties seen in Table 1, *NiNi* + *1* shows one 2-permutation of nucleotides positioned at sequence L, location *i* and *i+1*, and one physicochemical element is denoted as *P*_*j*_.

### *k-*Gram

The k-gram (Wu et al., 1992; Nanni, 2005) contains a pair of values (*v*,*c*), where *v*is an attribute and *c*is the number of occurrences of this function. The *v*is described as a mixture of several nucleotide units to evaluate the DNA sequence, and c is the number of variations in the series. For example, v belongs to the combined collection of 2 nucleotides to describe a DNA sequence with 2-gram, and *c* is the number of occurrences of each combination in the entire sequence.

k-gram is used to retrieve the features from the sequences, and the list *G* of a combination of nucleotides can be represented as follows.

(2) $$G=G_{1}\,\cup G_{2}$$

$$=\left\{N_{i}\right\}\,\cup \left\{N_{i}\,N_{j}\right\}$$

={A,C,G,T,A⁢A,A⁢C,…,T⁢T}

where *G*_*1*_ has 4 features with 1-gram, *G*_*2*_ has 16 features with 2-gram, *N*_*i*_*N*_*j*_ ∈ {*A*,*C*,*G*,*T*}.

Using k-gram to reflect DNA fragments (20 features), we can use simple statistical methods to obtain clear and intuitive sequence knowledge. When two segments are similar and have an additional related function, they may be more compatible in composition. The amount of each nucleotide in a section and their combinations indicates its composition directly.

### Multivariate Mutual Information

In many previous works (Cerf and Adami, 1998; Nanni and Lumini, 2006; Cao and Xiong, 2014), multivariate mutual information (MMI) was used to extract features from sequence data. Thus, MMI can also represent the nucleotide sequence. Inspired by previous research (Caragea et al., 2012; Ding et al., 2016), we suggest an advanced procedure for collecting nucleotide sequence features.

We first describe a set of 2-tuple nucleotide composition *T*_*2*_ and a set of 3-tuple nucleotide composition *T*_*3*_ to use multivariate shared knowledge on a DNA sample.

(3) $$T_{2}=\left\{A\,A,A\,C,A\,G,A\,T,C\,C,C\,G,C\,T,G\,G,G\,T,T\,T\right\}$$

(4) $$T_{3}=\left\{\begin{array}{c} A\,A\,A,A\,A\,C,A\,A\,G,A\,A\,T,A\,C\,C,A\,C\,G,A\,C\,T,A\,G\,G,A\,G\,T,A\,T\,T,\\ C\,C\,C,C\,C\,G,C\,C\,T,C\,G\,G,C\,G\,T,C\,T\,T,G\,G\,G,G\,G\,T,G\,T\,T,T\,T\,T \end{array}\right\}$$

Multivariate mutual information is not linked to nucleotide order in a tuple, so if two tuples have different orders but the same constant, they might have the same details and be classified as one tuple type. By *T*_*2*_ and *T*_*3*_, we can see that there is no structure with various order tuples, with 10 elements in *T*_*2*_ and 20 elements in *T*_3_.

For the elements in *T*_*2*_, we describe 2-tuple mutual information as follows:

(5) $$I\,\left(N_{1}\,N_{2}\right)=f\,\left(N_{1},N_{2}\right)\,l\,n\,\frac{f\,\left(N_{1},N_{2}\right)}{f\,\left(N_{1}\right)\,f\,\left(N_{2}\right)}$$

For the elements in *T*_*3*_, we describe 3-tuple mutual information as follows:

(6) $$I\,\left(N_{1}\,N_{2}\,N_{3}\right)=f\,\left(N_{1},N_{2}\right)\,l\,n\,\frac{f\,\left(N_{1},N_{2}\right)}{f\,\left(N_{1}\right)\,f\,\left(N_{2}\right)}+\frac{f\,\left(N_{1},N_{3}\right)}{f\,\left(N_{3}\right)}\,l\,n\,\frac{f\,\left(N_{1},N_{3}\right)}{f\,\left(N_{3}\right)}-\frac{f\,\left(N_{1},N_{2},N_{3}\right)}{f\,\left(N_{2}\right)\,f\,\left(N_{3}\right)}\,l\,n\,\frac{f\,\left(N_{1},N_{2},N_{3}\right)}{f\,\left(N_{2}\right)\,f\,\left(N_{3}\right)}$$

For a specific section, *f*(*N*_*i*_) is the occurrence of nucleotide *N*_*i*_ in this section, as *f*(*N*_*i*_,*N*_*j*_) and *f*(*N*_*i*_,*Nj*,*N*_*l*_) are the occurrences of 2-tuple and 3-tuple, correspondingly. *M*was used to represent the feature sets obtained from multivariate mutual information (30 features) and is defined as follows:

(7) M={I⁢(A⁢A),I⁢(A⁢C),…,I⁢(T⁢T),I⁢(A⁢A⁢A),I⁢(A⁢A⁢C),…,I⁢(T⁢T⁢T)}

### Discrete Wavelet Transform

**FIGURE 1:**
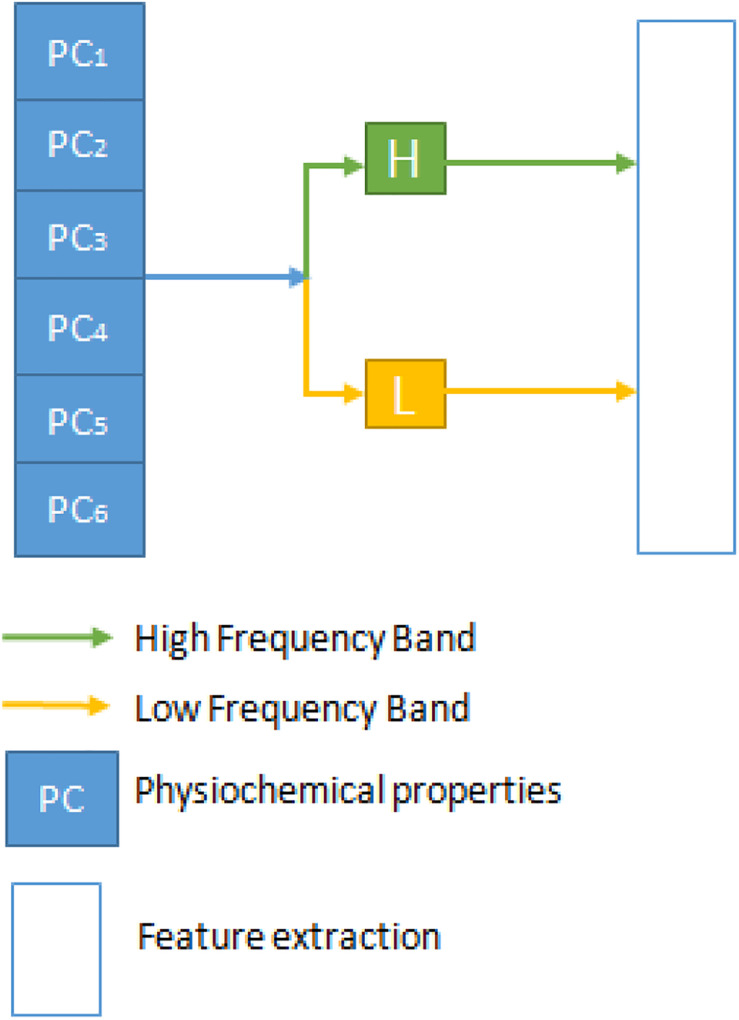
The discrete wavelet transform process.

Discrete Wavelet Transformation (DWT) is a transformation process where there are discreetly sampled wavelets that can collect both the details on information and frequency about location. This transition is a signal projection onto the function of a wavelet (Shensa, 1992). A rational and reliable model was recently built using wavelet packet decomposition and machine learning methods to predict the interspecies transmission of avian influenza virus from avian to human (Qiang and Kou, 2019). Once applied to DNA sequence review, DWT will degrade the nucleotide sequence’s physicochemical properties into a collection of coefficients at multiple resolutions. Furthermore, it also extracts the noise details from the high-passage profiles (Shen et al., 2017; Wang et al., 2017). Figure 1 is a 1-level separate transformation of the wavelet. Where PC stands for physiochemical properties, in total, there are six physicochemical properties. The details can be divided into a high-frequency band with noisier information and a low-frequency band with more reliable signals at each level and should be converted at the next stage.

**FIGURE 2:**
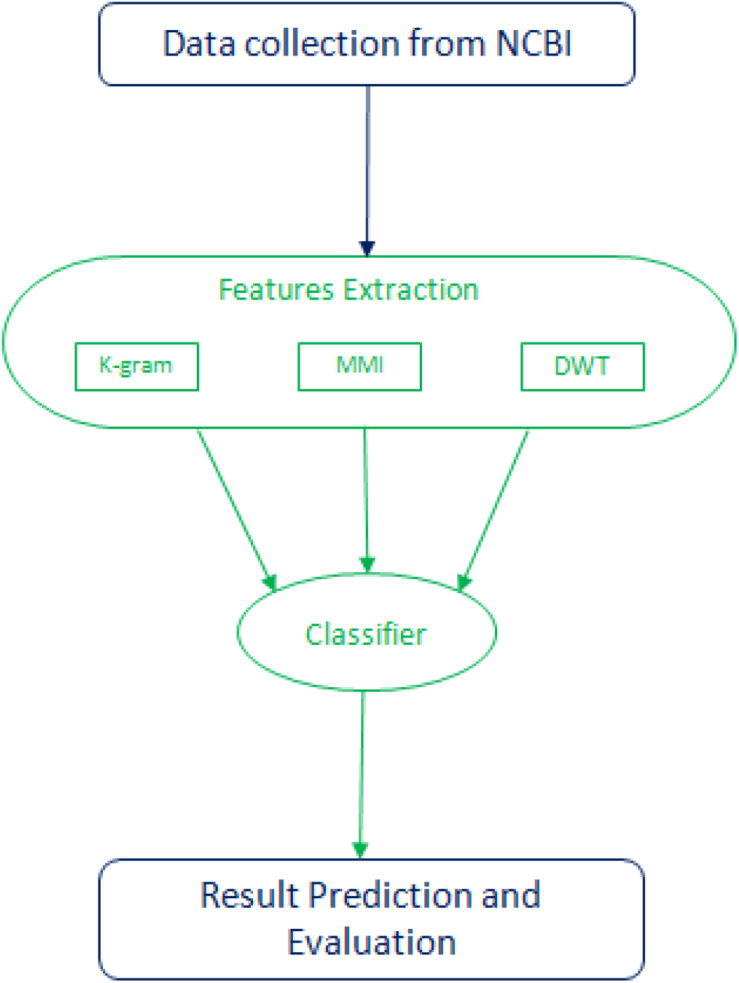
Flow chart of the methodology followed in this study.

Both high and low-frequency band signals are separated at each point of DWT. They measure each band’s average, mean, and standard deviation values. The first five components contain more critical information in the compact low-frequency band to reflect the series. Then we can get 4 + 4 + 5 features from each DWT stage, and the entire transformation process has 13 features. Within the physical property matrix PC, using a 1-level DWT method, we can extract 13 features. These six physicochemical characteristics can have 78 features. To denote this vector of the DWT function, we use the symbol D. Mean, Standard deviation, and average log of all six physicochemical characteristics are also taken separately as features adding 18 features. Figure 2 illustrates the flow chart of the methodology followed in this study. A combination of three feature selection methods, k-gram, Multivariate Mutual Information, and discrete wavelet transformation, were used to convert sequence information into feature vectors.

### Model Definitions

For the classification of Avian Influenza A virus subtype, we implemented several ML models for comparison. For the ML models, we applied Decision Tree, K Nearest Neighbor (kNN), Support Vector Machine, and Naïve Bayes for classification; the Naive Bayes classifier (John and Langley, 1995; Mitchell, 1997; Yu, 2001) is particularly suited to high-dimensional datasets based on the so-called Bayesian theorem. Given its apparent simplicity, this approach can also outperform more sophisticated classification systems (Huang et al., 2003). A decision tree is a method used to solve both classification and regression tasks for supervised automated learning. It derives rules from a list of objects defined in a class by a set of attributes. It is easy to grasp the derived laws since they can be visualized as a tree-like network (Kamiński et al., 2017). The kNN method (Cover and Hart, 1967) for supervised learning classification is one of the simplest instance-based learning algorithms. The classification is based on the consensus between the classes of the unknown entity’s closest k neighbors. The SVM classifier is statistically reliant on the Vapnik-Chervonenkis (VC) dimension (Haykin, 2009; Vapnik, 2013) and supports the soft margin hypothesis. It uses sequential minimal optimization (SMO) to solve the optimization problem. The issue is divided by SMO into a set of as minimal as possible sub-problems, which are then analytically solved. The aim is to differentiate groups from hyperplanes.

## Results

### Evaluation Criteria

To test the accuracy in classification of Avian Influenza A Virus, four statistical measurements were used to define its efficiency and performance as follows:

$$A\,c\,c\,u\,r\,a\,c\,y=\frac{\sum_{i=1}^{l}\frac{T_{P\,i}+T_{N\,i}}{T_{P\,i}+T_{N\,i}+F_{P\,i}\,F_{N\,i}}}{l}$$

$$P\,r\,e\,c\,i\,s\,i\,o\,n=\frac{\sum_{i=1}^{l}T_{P\,i}}{\sum_{i=1}^{l}T_{P\,i}+F_{P\,i}}$$

$$R\,e\,c\,a\,l\,l=\frac{\sum_{i=1}^{l}T_{P\,i}}{\sum_{i=1}^{l}T_{P\,i}+F_{N\,i}}$$

$$F\,1\,s\,c\,o\,r\,e=2\,\frac{P\times R}{P+R}$$

where true positive (TP) and true negative (TN) are total sequences that were predicted correctly as per their respective influenza A virus classes. While false positive (FP) and false negative (FN) are the numbers of mispredicted sequences according to respective influenza A virus classes. A total number of classes are denoted by *l*.

### Performance of Different Classifiers Using Our Method

The results of four different classifiers, Naïve Bayes, Decision Tree, K nearest neighbor (KNN), and Support Vector Machine (SVM), were compared using our method of feature selection. Our method involves the combination of three feature selection methods, i.e., N-gram, Multivariate Mutual Information, and Discrete Wavelet Transformation. This comparison is based on the 30% dataset separated from the original dataset for testing purposes. The results are listed in Table 2. Among the four classifiers, Decision Tree was the best, and Precision, Recall, F1 score, and Accuracy were 0.9514, 0.9535, 0.9524, and 0.9571, respectively. Decision Tree had the greatest accuracy over the other three classifiers using our method.

**TABLE 2:** Performance of classifiers.

Classifier	Precision	Recall	F1 score	Accuracy
**Naïve Bayes**	0.7355	0.7662	0.7366	0.7438
**Decision Tree**	0.9514	**0.9535**	0.9524	**0.9571**
**SVM**	0.9256	0.8694	0.8928	0.8671
**KNN**	**0.9595**	0.9534	**0.9562**	0.9485

*Bold digits are greatest values of each evaluation criteria.*

The Precision of the Decision Tree is 0.9514, which is slightly lower than that of KNN. The recall of the Decision Tree is 0.9535, which is better than the other classifiers. The F1 score of Decision Tree is 0.9524, which is also slightly lower than KNN, which is 0.9562. Still, the Decision Tree’s overall accuracy at 0.9571 is more significant than all other classifiers, while Naive Bayes, SVM, and KNN have 0.7438, 0.8671, and 0.9485 accuracies, respectively. Through these findings, we can see that our approach has greater accuracy and has produced more precise results.

As there is very little difference between Decision Tree and KNN we further used log loss function to find out the best classifier. The log loss function measures the efficiency of a classification model with a probability value varying from 0 to 1. If the expected value diverges from the real mark, the value of the log loss function increases. As shown in Table 3, Decision tree has a log loss value of 0.51 and KNN has 0.94, which shows that decision tree has performed better.

**TABLE 3:** Log loss function values of decision tree and KNN.

CLASSIFIER	LOG LOSS
Decision Tree	**0.51**
KNN	0.94

*Bold digits are greatest values of each evaluation criteria.*

### Feature Analysis

The involvement of every feature of the classifier is not similar. We performed tests using various combinations of features to assess each feature’s value and tested the qualified classifier. We used the Decision Tree because it was the best amongst other classifiers. The same dataset used for testing purposes was selected to perform this experiment.

The results of the experiments are given in Table 4. Initially, each feature set was used to train the classifier separately and use the testing data for evaluation. The k-gram records the presence and combination of a nucleotide. It could have 20 features. The simplest way to represent a nucleotide sequence is through these features. The classifier achieved 0.9161 on Precision using this 20-D element, 0.9175 on Recall, 0.9165 on F1 score, and 0.9114 on Accuracy. The MMI was able to obtain 30 features. They replicate the shared information in sequence. This form of representation is somewhat more composite than the k-gram, and it contains more important information. The classifier provided more substantial results with this 30-D feature than k-gram, with a gain of 0.9380 in accuracy.

**TABLE 4:** The performance of our method by using different features.

Features	Precision	Recall	F1 score	Accuracy
**k-gram**	0.9161	0.9175	0.9165	0.9114
**MMI**	0.9437	0.9410	0.9421	0.9380
**Combination 1**	**0.9469**	0.9446	0.9455	**0.9466**
**DWT**	0.9149	0.9159	0.9152	0.9071
**Combination 2**	0.9452	0.**9457**	**0.9466**	0.9445
**Combination 3**	0.9342	0.9395	0.9366	0.9438

*Combination 1 are features of 50-D, including k-Gram and MMI. Combination 2 are features of 126-D, including MMI and DWT. Combination 3 are features of 116-D, including N-Gram and DWT. Bold digits are greatest values of each evaluation criteria.*

Among the three individual feature sets, MMI’s 30-D feature trained the best classifier with precision reaching 0.9437, and the Recall, F1 score, and Accuracy were 0.9410, 0.9421, and 0.9380, respectively. DWT has not performed well individually as compared to the other classifiers. The combination of k-gram and MMI with 50-D features was the best combination of Precision methods at 0.9469, Recall 0.9446, F1 score 0.9455, and Accuracy 0.9466.

**FIGURE 3:**
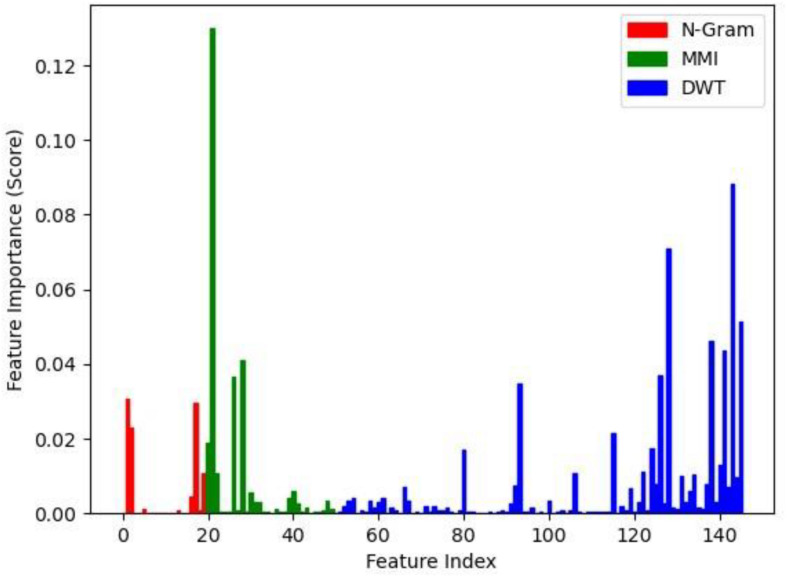
The importance score of each feature.

Figure 3 shows the effect of all items of a single function. There are overall 146 bars in this chart. The index of every feature is represented on the *x*-axis. The score of feature importance calculated using different feature selection methods implemented on the Decision Tree classifier is represented on the *y*-axis. It was seen that they were of varying significances to the classifier, and clearly, MMI had the most significant effect (the green color bars).

## Discussion

Avian influenza A Virus is major concern for both human and animal health (Guan et al., 2010; Suarez, 2010). The re-assortment and frequent HA gene mutations cause antigenic shift and drift of affected viruses, respectively (World Health Organization, 2011; Mak et al., 2012). Moreover, these events cause the rapid development of phylogenetic diversification that requires the subtyping and cluster classification for molecular epidemiological analysis (Altschul et al., 1990). Previously, no specific analysis was performed only on Avian influenza Viruses. It was seen through experimental results that our approach successfully extracts useful information from the DNA sequence and physical structure, particularly the combination of three feature extraction methods presented in this paper. The method proposed can utilize the benefit of the Avian Influenza Virus sequence’s original structure. The high precision of our approach’s predictive results indicates that the sequence of virus subtypes around the candidate site is necessary for prediction. After analyzing the feature performance experiment, it was found that DWT had more input to the classifier than k-gram and MMI methods. They are generating features from physicochemical properties. So, whether or not the physical structure around it affects an HPAIV in a very significant way.

A method’s running time on large data sets is a significant feature in assessing its feasibility. In order to get an intuitive grasp of our method’s computational complexity, it was applied via Python script and was executed on a P700 computer from Think Station. This machine contains two Intel R Xeon RE5 CPUs with 12 core and 320 G RAM. The CPU rate of the clock was 2.40 GHz. This software explicitly used one core and fewer than 10 G ram. We approximately calculated the runtime of each method of feature extraction, as described in Table 5. From these results, we can see that the k-gram algorithm was the shortest, and the cycle time is 2.00 s. The DWT method was more complicated because it took more than 45.23 s for execution. This shows that k-gram was far less complicated. MMI was strong compared with DWT, and its runtime was 8.06. The total execution time of our method was 59.70.

**TABLE 5:** Running time of all feature extraction methods.

	K-GRAM	MMI	DWT	COMBINE
**Running Time(s)**	2.00	8.06	45.23	59.70

Using our method, the Decision Tree algorithm could learn 550 sequences per second when training the classifier. This is very fast. This means our method could complete the training process in 10 min for a dataset size of one million dataset. This low difficulty makes our approach ideal for functional purposes.

## Conclusion

A novel method was proposed in this paper for predicting subtypes of Avian Influenza Virus. We developed and implemented a combination of three methods for extracting sequence and physical structure features from HA and NA subtype sequences. K-gram, discrete wavelet transformation, and multivariate mutual information were the three methods of extraction of features. We also analyzed the significance of each of these methods. The results of four different classifiers, Decision Tree, Support Vector Machine (SVM), Naïve Bayes, and K nearest neighbor (KNN), were compared using our feature selection method. This comparison is based on the 30% dataset separated from the original dataset for testing purposes. Among the four classifiers, Decision Tree was the best, and Precision, Recall, F1 score, and Accuracy were 0.9514, 0.9535, 0.9524, and 0.9571, respectively. Decision Tree had significant improvements as compared to the other three classifiers using our method. Our method effectively predicted the Avian influenza Virus subtype, based on the evaluation value and comparison.

## Data Availability Statement

The datasets presented in this study can be found in online repositories. The names of the repository/repositories and accession number(s) can be found below: https://www.ncbi.nlm.nih.gov/genomes/FLU/Database/nph-select.cgi?go=database.

## Author Contributions

FH, FK, NF, D-QW, and SS designed and implemented the manuscript. AF, AA, AK, and FH carried out the manuscript writing. D-QW, AK, and AF revised the manuscript. All authors contributed to the article and approved the submitted version.

## Conflict of Interest

The authors declare that the research was conducted in the absence of any commercial or financial relationships that could be construed as a potential conflict of interest.

## Footnotes

1 http:/www.fludb.org
